# Cellular Heterogeneity of Pancreatic Stellate Cells, Mesenchymal Stem Cells, and Cancer-Associated Fibroblasts in Pancreatic Cancer

**DOI:** 10.3390/cancers12123770

**Published:** 2020-12-15

**Authors:** Yoshiaki Sunami, Johanna Häußler, Jörg Kleeff

**Affiliations:** Department of Visceral, Vascular and Endocrine Surgery, Martin-Luther-University Halle-Wittenberg, University Medical Center Halle, 06120 Halle, Germany; johanna.haeussler@uk-halle.de (J.H.); joerg.kleeff@uk-halle.de (J.K.)

**Keywords:** pancreatic cancer, cancer-associated fibroblasts, pancreatic stellate cells, mesenchymal stem cells, cancer-restraining cancer-associated fibroblast, cellular heterogeneity

## Abstract

**Simple Summary:**

Cancer-associated fibroblasts, which are derived from several cell types such as pancreatic stellate cells or mesenchymal stem cells, play a major role in the progression and drug resistance of pancreatic cancer. Targeting cancer-associated fibroblasts can, therefore, be a promising therapeutic strategy. However, several studies show that deletion of cancer-associated fibroblasts may also have tumor-promoting effects. These contrasting observations suggest that cancer-associated fibroblasts exhibit functional heterogeneity in pancreatic cancer.

**Abstract:**

Pancreatic cancer is projected to become the second deadliest cancer by 2030 in the United States, and the overall five-year survival rate stands still at around 9%. The stroma compartment can make up more than 90% of the pancreatic tumor mass, contributing to the hypoxic tumor microenvironment. The dense stroma with extracellular matrix proteins can be a physical and metabolic barrier reducing therapeutic efficacy. Cancer-associated fibroblasts are a source of extracellular matrix proteins. Therefore, targeting these cells, or extracellular matrix proteins, have been considered as therapeutic strategies. However, several studies show that deletion of cancer-associated fibroblasts may have tumor-promoting effects. Cancer-associated fibroblasts are derived from a variety of different cell types, such as pancreatic stellate cells and mesenchymal stem cells, and constitute a diverse cell population consisting of several functionally heterogeneous subtypes. Several subtypes of cancer-associated fibroblasts exhibit a tumor-restraining function. This review article summarizes recent findings regarding origin and functional heterogeneity of tumor-promoting as well as tumor-restraining cancer-associated fibroblasts. A better understanding of cancer-associated fibroblast heterogeneity could provide more specific and personalized therapies for pancreatic cancer patients in the future.

## 1. Introduction

Pancreatic ductal adenocarcinoma (PDAC) is a devastating disease with an unfavorable outcome. Currently, pancreatic cancer is the seventh leading cause of global cancer deaths in industrial countries [1], and is projected to become the second deadliest cancer by 2030 in the United States [2]. The overall five-year survival rate stands still at 9% [3]. A number of studies have shown significant progress in survival of pancreatic cancer patients by combination chemotherapies [4,5,6,7,8]. However, pancreatic cancer still exhibits remarkable resistance to radiotherapy and chemotherapy, due to genetic and epigenetic alterations, together with a complex and desmoplastic tumor microenvironment [9,10].

The tumor microenvironment includes immune cells, cancer-associated fibroblasts (CAFs), which are, in part, derived from pancreatic stellate cells (PSCs) and endothelial cells, resulting in an abundant collection of inflammatory cytokines and chemokines, growth factors, and components of the extracellular matrix (ECM). The stromal compartments can make up more than 90% of the pancreatic tumor mass, which generates the hypoxic tumor microenvironment [9]. ECM proteins are produced by activated PSCs and CAFs, which support pancreatic fibrogenesis and hypoxia [11]. The dense stroma with ECM proteins can be a physical and metabolic barrier reducing therapeutic efficacy [12]. The hypoxic microenvironment leads to activation of angiogenesis, metastasis, and metabolic reprogramming, where hypoxia inducible factors (HIFs) play a central role [12]. To that end, targeting CAFs/activated PSCs or disrupting ECM are promising therapeutic strategies. The data have, however, suggested that deletion of CAFs also have tumor-promoting effects. Deletion of alpha-smooth muscle actin (α-SMA, a marker of myofibroblasts)-positive cells in a pancreatic cancer mouse model (*Ptf1-Cre*; *lox-stop-lox-Kras^G12D/+^*; *Tgfbr2^lox/lox^*) leads to invasive, undifferentiated tumors that are more hypoxic and reduces animal survival [13]. Deletion of α-SMA-positive cells also diminishes animal survival in another mouse model called KPC (*Pdx1-Cre*; *lox-stop-lox-Kras^G12D/+^*; *lox-stop-lox-Trp53^R172H/+^*) [13]. KPC and *Ptf1-Cre*; *lox-stop-lox-Kras^G12D/+^*; *Tgfbr2^lox/lox^* are mouse models that spontaneously develop pancreatic cancer recapitulating clinical and histopathological features of the human disease [13,14]. Myofibroblast-depleted tumors display increased invasion associated with intra-tumoral hypoxia in *Ptf1-Cre*; *lox-stop-lox-Kras^G12D/+^*; *Tgfbr2^lox/lox^* mice [13]. Sonic hedgehog (Shh) signaling is known to drive formation of a fibroblast-rich desmoplastic stroma [15,16]. Inhibition of Shh signaling with IPI-926, which is a semisynthetic derivative of cyclopamine, enhances delivery of gemcitabine in KPC mice [17]. However, conditional deletion of Shh (*Shh^lox/lox^*) reduces stromal content, but tumors are more aggressive, undifferentiated, and display increased vascularity in the *Pdx1-Cre*; *lox-stop-lox-Kras^G12D^*; *Trp53^lox/+^* pancreatic cancer mouse model [18]. Furthermore, a clinical study (NCT01130142) that combined chemotherapy with IPI-926 and gemcitabine showed a shortened patient overall survival than gemcitabine treatment alone and was, therefore, terminated prematurely [19]. These studies imply that neither removal of cells based solely on α-SMA positivity nor abrogation of Shh expression or its pharmacologic inhibition are effective in limiting tumor aggressiveness. The fact that these treatments limit stromal development imply a restrictive role of some kinds of CAF without proving the absence of a tumor-promoting role in other kinds.

## 2. Origin and Functional Heterogeneity of Cancer-Associated Fibroblasts in Cancer

**Figure 1:**
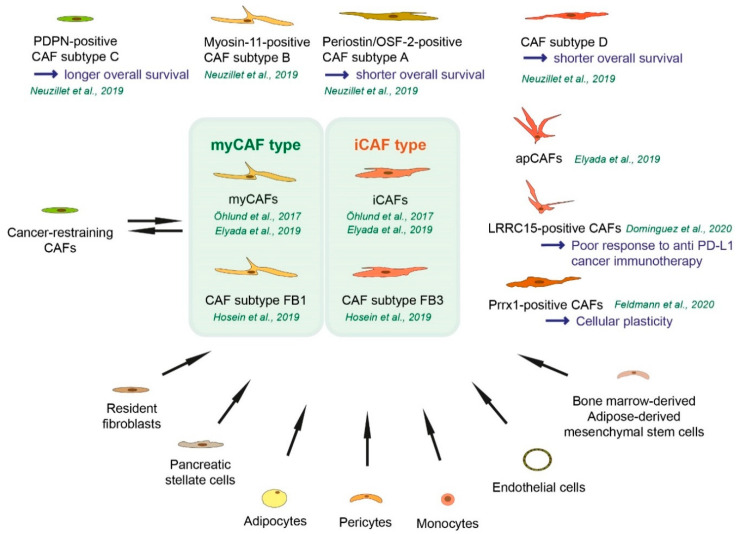
Origin and functional heterogeneity and classification of cancer-associated fibroblasts. Non-fibroblast lineage cells such as monocytes or endothelial cells can become a part of cancer-associated fibroblasts (CAFs). CAFs represent very heterogeneous subpopulations with different functions in tumor-promoting and tumor-restraining manner. Tumor-promoting CAFs can be converted to tumor-restraining CAFs and vice versa.

In general, activated fibroblastic cells in the tumor microenvironment of solid cancers that have a phenotype, function, or location distinct from quiescent fibroblasts are considered as CAFs [20]. CAFs are characterized by their diverse origins. Resident fibroblasts and especially PSCs are major sources of CAFs in pancreatic cancer [21,22,23]. Furthermore, adipocytes, pericytes, monocytes, endothelial cells, and bone marrow-derived or adipose-derived mesenchymal stem cells (MSCs) can differentiate into CAFs (Figure 1) [24,25,26,27]. Cultured MSCs themselves exhibit a high cellular plasticity and capacity to undergo osteogenic, adipogenic, myogenic, and chondrogenic differentiation [28].

In the healthy pancreas, PSCs are in the quiescent state. The most consistent marker of quiescent PSCs is the presence of cytoplasmic vitamin A droplets [22]. In addition, PSCs can be identified by the expression of desmin, glial fibrillary acidic protein (GFAP), and acetylcholine receptors (Table 1) [20]. Extracellular signals, such as transforming growth factor (TGF)-β, tumor necrosis factor (TNF)-α, interleukins, and oxidative stress modulate PSC activation (Table 2) [22]. Besides ECM proteins, activated and transformed PSCs produce several stimulators such as platelet-derived growth factor (PDGF), connective tissue growth factor (CTGF), and epidermal growth factor (EGF), supporting cancer cell proliferation [22]. PSCs can undergo autophagy to support pancreatic fibrosis and tumor growth [29]. Autophagy of PSCs is associated with shorter survival of pancreatic cancer patients [29]. Pancreatic cancer cells stimulate autophagy in PSCs [30] and the alanine released from PSCs undergoing autophagy is utilized by pancreatic cancer cells [30]. It was discovered in the inducible oncogenic KRAS (Kirsten Rat Sarcoma) mouse model (*Ptf1-Cre*; *Rosa26-rtTa*; *TetO-Kras^G12D^*) in which pancreatic tumor cells regulate PSCs non-cell-autonomously by secreting factors including SHH protein [31]. Pancreatic cancer cells are further modulated by signal transduction of reciprocal signals from activated PSCs. For example, signals from SHH-activated PSCs are transduced through the insulin growth factor 1 receptor (IGF1R) on tumor cells (Table 2) [31].

It has been shown that, in an inflammation-induced gastric cancer model with *Helicobacter felis* infection, at least 20% of CAFs originate from bone marrow and derive from MSCs. MSC-derived CAFs that are recruited to the dysplastic stomach express IL-6, Wnt5a, and bone morphogenetic factor 4 (BMP4) (Table 1 and Table 2) [32]. In the case of breast cancer, bone marrow-derived MSCs (BM-MSCs) cause the cancer cells to increase their metastatic potency by stimulating de novo secretion of CCL5 (also called RANTES) secreted by BM-MSCs. CCL5 acts as a reciprocal signal to the breast cancer cells to enhance their motility, invasion, and lung metastasis (Table 2) [33]. In another study, it has been shown that BM-MSCs are recruited to primary breast cancer as well as to lung metastasis. BM-MSC-derived CAFs (BM-CAFs) are a substantial source of CAFs in the tumor microenvironment. They support tumor growth and enhance angiogenesis via Clusterin expression. Resident CAFs do, but BM-derived CAFs do not express PDGFRα. Therefore, the recruitment of BM-CAFs results in a decrease in the ratio of PDGFRα-expressing CAFs. A decrease in PDGFRα expression in breast cancer patients is associated with shorter overall survival [38]. CD44^+^/CD73^+^/CD90^+^/CD49α^+^ MSCs are detected in pancreatic cancer and termed cancer-associated MSCs (CA-MSCs) [34]. Pancreatic CA-MSCs, but not non-MSC CAFs, promote tumor cell growth, invasion, and metastasis [34]. CA-MSCs secrete granulocyte-macrophage colony-stimulating factor (GM-CSF), and pancreatic cancer cells express GM-CSF receptor. CA-MSC-derived GM-CSF is required for pancreatic cancer cell invasion and metastasis (Table 2) [34]. The therapeutic potential of MSCs has been studied in several clinical trials including studies targeting gastrointestinal, lung, and ovarian cancer [39]. The TREAT-ME1 study (NCT02008539) used bone marrow-derived MSC-delivery of Herpes simplex virus type 1 thymidine kinase (HSV-TK) under the control of the *CCL5*-promotor. After cell delivery, a cytotoxic drug ganciclovir is administered, which is phosphorylated and activated by HSV-TK for cancer cell death [40]. A Phase 1 study (NCT02530047) to test the safety of interferon β (IFN-β)-secreting MSCs has been completed. A Phase 1/2 study (NCT03298763, TACTICAL trial) recruits lung cancer patients using MSCs to deliver the TNF-related apoptosis-inducing ligand (TRAIL). An inhibitory effect of fibroblasts on cancer cell proliferation has been proposed and named cancer-restraining CAFs [41], which is further discussed below (see Section 5). There is evidence to suggest that CAFs can arise from adipocytes and adipose-derived MSCs in several solid tumors. In the case of breast cancer, adipocyte cells co-cultured with cancer cells are differentiated into fibroblast-specific protein 1 (FSP-1)-positive, but not α-SMA-positive, CAFs [42]. Adipose-derived MSCs are converted into CAFs in the presence of breast cancer cell-derived factors. Adipose-derived MSCs (AD-MSCs) from obese donors express higher levels of *ACTA2* (coding α-SMA), VEGF, fibroblast activation protein (*FAP*), *S100A4* (FSP), and chondroitin surface proteoglycan 4 *CSPG4* (also known as NG2) when compared to AD-MSCs from lean donors [35]. Ovarian cancer cells induce differentiation of AD-MSCs into α-SMA and FAP-positive CAFs [43]. AD-MSCs can differentiate into distinct CAF subtypes dependent on different co-culture systems [26]. Distinct CAF subtypes are discussed in Section 4. CAFs are characterized by their diverse origins and differentiation into distinct CAF subtypes, which depends on the proximity to other cell types.

## 3. Fibroblast and Cancer-Associated Fibroblast Markers

In a healthy pancreas, distinct fibroblast populations and mesenchymal heterogeneity are observed [44]. Two different populations of pancreatic fibroblasts have been characterized, by the expression of transcription factors GLI1 and HOXB6 [45]. The transcription factor GLI1 is a terminal effector of the Hedgehog pathway. Hedgehog signaling is active during embryonic development but largely suppressed in the adult [46]. Yet, a small population of GLI-positive fibroblasts can be observed in the healthy pancreas [47]. Activation of GLI transcription factors is required for formation of oncogenic KRAS-dependent pancreatic cancer in mice [48]. The study used *Ptf1-Cre*; *lox-stop-lox-Kras^G12D^*; *Rosa26-Gli3T* mice, where GLI3 acts as a dominant repressor of *Gli* transcription [48]. Furthermore, GLI1 accelerates KRAS-initiated pancreatic tumorigenesis in global *Gli1* knock-in mice (*Ptf1-Cre*; *lox-stop-lox-Kras^G12D/+^*; *Rosa26-Gli1*) [48]. HOX6 paralogs (HOXA6, HOXB6, and HOXC6) are expressed in the mesoderm of the developing pancreas and mesenchyme but not in the endoderm [49]. GLI1 and HOXB6 are expressed in largely distinct populations in the healthy pancreas. Lineage-tracing experiments revealed that GLI1-positive cells, but not Hoxb6-positive cells, which proliferated and promoted the fibrotic reaction in pancreatic cancer. In a pancreatic cancer mouse model (*Ptf1-Cre*; *frp-stop-frp-Kras^G12D/+^*; *Gli1^CreERT^*), lineage-traced, GLI1-positive cells account for a little less than half of the total myofibroblast population [45].

In response to tissue injury or stimuli, quiescent fibroblasts are activated to become a normal activated fibroblast (NAFs) to facilitate repair and regeneration. The NAFs gain expression of α-SMA and vimentin. The NAFs further gain contractile properties along with enhanced ECM production, remodeling, and cytoskeletal rearrangement. With persisting injury or with the development of cancer lesions, activated fibroblasts further gain enhanced proliferation properties and form a functionally diverse population as fibrosis-associated fibroblasts (FAFs) or CAFs, respectively [28].

A number of different markers can identify activated fibroblasts including α-SMA, desmin, FAP, fibroblast-specific protein (FSP1, also known as S100A4), PDGFRα, PDGFRβ, podoplanin (PDPN), and vimentin [20,28]. However, proposed activated fibroblast markers are not specific for fibroblasts or activated fibroblasts [28]. For example, FSP1 has been identified in an inflammatory subpopulation of macrophages in the liver [50]. FAP is widely considered one of the most reliable CAF markers. However, only a certain subpopulation of CAFs expresses FAP and it is completely absent in other tumor fibroblast subpopulations [51]. A Phase 2 trial of FAP inhibition using the humanized monoclonal antibody sibrotuzumab failed to slow tumor progression in colorectal cancer patients (NCT02198274) [10,52]. α-SMA has been considered as a key marker for identifying CAF populations. High expression of α-SMA in CAFs is associated with shorter overall survival in breast and colon cancer [53,54]. However, the *ACTA2* gene (coding α-SMA) expression in patients-derived CAFs and *Acta2* gene and α-SMA expression in murine (KPC mice)-derived CAFs or primary PSCs drop when cultured with conditioned media from organoids derived from pancreatic cancer patients or from organoids derived from KPC mice, respectively [36] (see Section 4.). Therefore, α-SMA cannot be used for all CAF populations. PDGFRs are expressed in several cancer types, fibroblasts, astrocytes, neuro-progenitors, and pericytes [55,56], but are more broadly expressed in fibroblasts than comparative markers like α-SMA [56]. PDPN is an O-glycosylated transmembrane glycoprotein that is expressed not only in CAFs but also observed in lymphatic endothelial cells, kidney podocytes, keratinocytes, mesothelial cells, and in several types of cancer. PDPN expression in CAFs is correlated with shorter overall survival of pancreatic cancer patients [57]. Vimentin is an intermediate filament protein involved in the formation of the cytoskeleton network [56]. Vimentin is highly expressed in fibroblasts, but also in smooth muscle cells, endothelial cells, and cancer cells [58].

As a marker of activated fibroblasts, Y397 phosphorylated focal adhesion kinase (FAK) has been considered. FAK is a key intracellular effector of ECM signaling, which gets activated upon ECM-induced integrin receptor activation. This leads to auto-phosphorylation of FAK at Y397 [59]. CAFs from pancreatic cancer patients exhibit an increase of phosphorylated Y397 FAK compared to controls. Higher levels of Y397-phosphorylated FAK are associated with shorter disease-free and overall survival of pancreatic cancer patients [60]. Since most markers are shared with other cell types, further identification of CAF subtype-specific markers are required. Furthermore, it is possible that a single marker identifies a range of distinct CAF subtypes that may have functionally opposing roles in cancer progression [58]. Therefore, it will be necessary to subdivide CAFs by the CAF subtype-specific markers or a combination of several markers.

## 4. Cancer-Associated Fibroblast Subtype Classification in Pancreatic Cancer

To characterize the heterogeneity of fibroblasts and to understand the molecular mechanism in pancreatic cancer immunity and progression, it has been important to investigate cell type-specific gene expression. It has been shown that the majority of fibroblasts in human pancreatic tumors as well as in tumors from KPC mice express FAP and low levels of α-SMA, whereas a subpopulation of FAP-positive cells exhibit elevated α-SMA expression (named as myo-fibroblastic CAFs “myCAFs”) [36]. CAFs co-cultured with organoids derived from pancreatic cancer patients secrete IL-6, IL-11, and leukemia inhibitory factor (LIF), and these ligands activate a signal transducer and activator of transcription 3 (STAT3) in organoids (Table 2) [36]. This cytokine-expressing CAF subpopulation with low α-SMA expression is named inflammatory CAFs (iCAFs) [36]. In pancreatic cancer, in patients and in KPC mice, myCAFs and iCAFs co-exist [36]. MyCAFs are located in the peri-glandular region, iCAFs are located more distantly from tumor cells and myCAFs in pancreatic cancer [36]. A cluster of genes including *Acta2* (coding α-SMA), *Vim*, *Ctgf*, *Col1a1*, *Col5a1*, and *Col6a1* are up-regulated in myCAFs, whereas interleukins *Il1*, *Il6*, *Il11*, and *Lif* as well as chemokines such as *Cxcl1*, *Cxcl2* are up-regulated in iCAFs (Table 3) (Figure 1) [36]. For the transcriptomic analysis, mouse quiescent PSCs, α-SMA^low^ IL-6^high^ mouse PSCs cultured in trans-well with KPC tumor organoids for iCAFs, and α-SMA^high^ IL-6^low^ PSCs for myCAFs, were used [36]. AD-MSCs (see Section 2) can differentiate into distinct CAF subtypes dependent on different co-culture systems, between direct contact co-culture with pancreatic cancer cells (cancer cells and AD-MSCs) are mixed or indirect contact with pancreatic cancer cells (cancer cells and AD-MSCs are separated by a trans-well membrane). Direct contact co-culture induces differentiation into myCAFs and iCAFs, while indirect co-culture induces differentiation into only iCAFs [26]. PSCs can differentiate into myCAFs and iCAFs when co-cultured with organoids derived from KPC mice [36]. These findings suggest that direct juxacrine interactions with cancer cells are required for myCAF formation [36].

In another study (single cell RNA sequencing) with surgically resected low-grade intraductal papillary mucinous neoplasms (IPMNs), high-grade IPMNs, and invasive pancreatic cancer, myCAFs and iCAFs are observed. The iCAF subpopulation is detected in invasive pancreatic cancer, and iCAFs exhibit elevated expression of *VIM*, *FAP*, *COL3A1*, *DES*, *IL6*, and *CXCL12* and reduced expression of *ACTA2* (coding α-SMA). The myCAF population is rare in low-grade IPMNs but is highly represented in high-grade IPMNs, and exhibit increased expression of *ACTA2* and reduced expression of *CXCL12* and *DES* [65]. Neuzillet and colleagues performed RNA sequencing and identified four different subtypes in primary isolated CAFs from pancreatic cancer patient specimens (named subtype A to D) (Figure 1). Periostin is considered as a subtype A biomarker and high expression of periostin is associated with shorter overall survival of pancreatic cancer patients. Subtypes B-D CAFs induce more cancer cell proliferation than subtype A CAFs [23]. Periostin, or osteoblast-specific factor 2 (OSF-2), is considered an important molecule in several diseases such as scar formation in myocardial infarction, fibrosis, or cancer cell migration [22,66]. Subtype B displays myogenic properties and myosin-11, which is a smooth muscle myosin, is selected as a subtype B biomarker [23]. Subtype C CAF-related marker is PDPN, which is a cell-surface mucin-like glycoprotein. Pancreatic cancer patients with the dominant subtype C CAFs show prolonged overall survival [23]. In another study, PDPN expression in CAFs correlated with shorter overall survival of pancreatic cancer patients [57] and a study suggests heterogeneity within the PDPN-positive stroma [63]. Patients with dominant subtype D CAFs show shorter overall survival [23]. In subtype D CAFs, *CXADR* (Coxachie virus and Adenovirus Receptor, also known as CAR), *MEOX* (Mesenchyme Homeobox), and *PLS1* are highly expressed [23]. CXADR is an essential regulator of cell growth and adhesion during development [67]. The MEOX family of transcription factors are important during mammalian embryogenesis and axial skeleton development [68], PLS1 (Plastin 1), also called fimbrin, is one of the most abundant actin-bundling proteins [69]. *CXADR* and *MEOX* are expressed in tumor cells, *PLS1*, is expressed in endothelial cells [23].

In addition to *KRAS* mutation, additional driver events are required for pancreatic cancer progression, where *TP53* and *CDKN2A* are the most commonly mutated genes [70]. Ink4a/p16 inhibits cell cycle progression through the G1/S checkpoint mediated by CDK4 and CDK6 [71]. To profile cell heterogeneity during different stages of pancreatic cancer progression in *Ptf1-Cre*; *lox-stop-lox-Kras^G12D/+^*; *Ink4a^lox/lox^* mouse model, the single-cell RNA-sequencing has been conducted [62]. In this study, there are three distinct molecular subtypes of fibroblasts in the healthy mouse pancreas, and two distinct populations of CAFs in advanced pancreatic cancer [62]. Distinct subtypes of CAFs are observed in another pancreatic cancer mouse model, *Ptf1-Cre*; *lox-stop-lox-Kras^G12D^*; *lox-stop-lox-Trp53^R172H/+^* [62]. The two CAF subpopulations exist across different advanced-stage pancreatic cancer mouse models, suggesting a consistent cell of origin [62]. A fibroblast subpopulation in healthy mouse pancreas in *Ptf1-Cre*; *lox-stop-lox-Kras^G12D/+^*; *Ink4a^lox/lox^* mice express chemokine *Cxcl14*, *Ptn*, and several genes associated with insulin-like growth factor (IGF) signalling such as *Igf1*, IGF-binding protein (IGFBP) family *Igfbp4*, and *Igfbp7* (called here FB1) [62]. The second subpopulation (FB2) express *Ly6a*, *Ly6c1*, which is a CCN family secreted matricellular protein member *Nov*, and *Pi16*. The third population (FB3) expresses several mesothelial markers such as *Lrrn4*, *Gpm6a*, *Nkain4*, *Lgals7*, and *Msln* as well as *Cav1*, *Cdh11*, and *Gas6* (Table 3) [62]. In early lesions, the FB2 subpopulation starts to move toward an FB1 expression profile. *Il6*, *Ccl2*, *Ccl7*, *Cxcl12*, and *Pgdfra* are expressed in FB1 subtype fibroblasts in the normal pancreas, as well as during the progression of pancreatic cancer [62]. The myofibroblast markers such as *Acta2* and *Tagln* are expressed in the FB3 subpopulation. These findings can support the presence of iCAFs (FB1) and myCAFs (FB3) in the pancreatic cancer mouse models (Figure 1) [62].

The FB3 fibroblast subpopulation expresses several major histocompatibility complex (MHC) class II-associated genes [62]. In another single-cell analysis, a subpopulation of CAFs that express MHC class II and CD74 is described and named antigen-presenting CAFs (apCAFs) [61]. It is not clear whether the FB3 CAF subpopulation can be divided into myCAFs and apCAFs. The apCAF subpopulation further expresses *Saa3* and *Slpi*, and the subpopulation detected in tumors of KPC mice as well as in pancreatic cancer patients. MHCII molecules of apCAFs have the capacity to present antigens to CD4^+^ T cells, but apCAFs lack the costimulatory molecules needed to induce T-cell proliferation, potentially contributing to immune suppression [61]. In this study, selected markers for each CAF subpopulation are summarized as follows: markers for myCAFs are *Tnc*, *Tgfb1*, *Thy1*, *Tagln*, *Col12a1*, and *Pdgfrb*. Selected markers for iCAF are *Clec3b*, *Col14a1*, *Gsn*, *Ly6c1*, and *Cxcl12*. Markers for apCAFs are *Slpi*, *Saa3*, *Cd74*, *H2-Ab1*, *Nkain4*, and *Irf5* (Table 3) [61]. Mechanistically, IL-1 induces LIF and activates JAK/STAT signaling to generate iCAFs (Table 2) [37]. TGFβ downregulates IL1R1 and promotes differentiation into myofibroblasts [37].

A recent study identified a leucine-rich repeat containing 15 (LRRC15)-positive CAF subpopulation within the PDPN-positive CAF population in pancreatic cancer mouse model (*Pdx1-Cre*; *lox-stop-lox-Kras^G12D/+^*; *p16/p19^lox/lox^*) [63]. The LRRC15-positive CAF subpopulation can be identified in pancreatic cancer patient specimens [63]. Mouse tumor spheroids cultured with LRRC-positive CAFs grow larger than those in media alone, suggesting that LRRC-positive CAFs can directly enhance tumor growth [63]. Elevated LRRC15 levels correlates with a poor response to anti-PD-L1 cancer immunotherapy in six immune-excluded cancer types (characterized by a dense stromal structure surrounding the tumor and the presence of most tumor-infiltrating cells in the stromal area, fewer in the tumor parenchyma), pancreatic cancer, bladder cancer, renal cell carcinoma, head and neck cancer, and non-small cell lung cancer (Figure 1) [63].

The paired-related homeobox (Prrx1) is considered a driver of cellular plasticity during pancreatic ductal development, acinar-to-ductal metaplasia (ADM) formation, and carcinogenesis [72]. In *Pdx1-Cre*; *lox-stop-lox-Kras^G12D/+^*; *Ink4a^lox/+^* murine pancreatic cancer and in pancreatic cancer patient specimens, Prrx1 is expressed in CAFs. High expression levels of Prrx1 in CAFs are associated with squamous subtypes of pancreatic cancer [64]. Patients with the squamous subtype have shorter survival (Median survival 30.0/23.7/25.6 months for abnormally differentiated endocrine exocrine, pancreatic progenitor, immunogenic subtypes, 13.3 months for the squamous subtype) [73]. Prrx1-deficient CAFs display a myCAF identity with increased expression of VIM, α-SMA, SPARC, and COL1A1 due to attenuated plasticity. Targeting Prrx1 can, therefore, be a treatment strategy by reducing CAF plasticity by forcing myCAF differentiation and the conversion of tumor-promoting to tumor-restraining CAFs (Figure 1) [64].

## 5. Cancer-Restraining Cancer-Associated Fibroblasts

It has been demonstrated that static normal fibroblasts suppress polyoma virus-transformed tumor cells [74], suggesting that an innate function of fibroblasts is to protect against tumorigenesis [75]. Subtypes of tumor-restraining CAFs and their specific marker proteins have not been well characterized. A recent study suggests that a CAF subtype expresses Meflin, a glycosylphosphatidylinositol-anchored protein encoded by the immunoglobulin superfamily containing a leucine-rich repeat gene (*ISLR*) [76]. Meflin has been considered as an MSC marker and expressed in MSCs, fibroblasts, pericytes, perivascular cells, and bone marrow [77]. Meflin expression in CAFs correlates with longer overall survival of patients with pancreatic cancer. Tumors in KPC mice with Meflin deficiency are larger and more proliferative than those developed in KPC mice with intact Meflin. Meflin knockout KPC mice show shorter overall survival than Meflin wild-type KPC mice. Meflin suppresses poor differentiation of pancreatic cancer [76]. Meflin is expressed by approximately 40% of FAP-positive CAFs and by the majority of CAFs expressing GLI1 [76]. As mentioned above, GLI1 accelerates KRAS-initiated pancreatic tumorigenesis in *Gli1* knock-in mice, suggesting that CAF heterogeneity may exist within GLI-positive fibroblasts (see Section 3).

CD271, also known as a neurotrophin receptor, nerve growth factor receptor, NGFR, or p75NTR, has been implicated in tumor growth [78]. CD271 has been identified as a marker of MSCs [79]. Expression of CD271 is also observed in hepatic and pancreatic stellate cells [80,81]. Histologically, CD271-positive CAFs/PSCs are observed on the edge rather than in the center of tumors [78]. Another study also shows that CD271 expression is higher in peripheral CAFs than in juxta-tumoral CAFs [82]. High stromal expression of CD271 is associated with longer overall survival of pancreatic cancer patients [78]. Intriguingly, CD271 is highly expressed in CAFs in regressive stromal compartments in neoadjuvant FOLFILINOX (folinic acid, 5-fluorouracil, irinotecan and oxaliplatin) treated pancreatic cancer patients [82]. Whether we can consider CD271 as a cancer-restraining CAF marker or not, and whether neoadjuvant chemotherapy can trigger the conversion of tumor-promoting to tumor-restraining CAFs or not, still needs to be clarified.

α-SMA has been considered as a candidate marker of tumor-restraining CAFs based on the observation in a pancreatic cancer mouse model [13] (see Section 1). However, several studies have shown that the number of α-SMA-positive CAFs correlates with shorter overall survival in esophageal and pancreatic cancer patients [83,84]. Since CAFs are a heterogeneous population in the tumor microenvironment, it is plausible to consider that the “stromal switch” involves the conversion of tumor-restraining CAFs to tumor-promoting CAFs [41]. The “stromal switch” can be drivers of cellular plasticity, such as Prrx1. Furthermore, it is important to identify such “stromal switches,” which would provide more specific therapies for pancreatic cancer patients. The specific markers of cancer-restraining CAFs need further identification and characterization.

## 6. Conclusions

CAFs represent very heterogeneous subpopulations, which can function in tumor-promoting as well as in a tumor-restraining manner. CAFs are derived in a wide variety of different cell types such as pancreatic stellate cells, bone marrow-derived, or adipose-derived mesenchymal stem cells, adipocytes, pericytes, monocytes, and endothelial cells, leading to and supporting CAF heterogeneity. The tumor microenvironment can also trigger the conversion from one CAF subtype to another CAF subtype. Further identification and characterization of CAF subtypes are required, but it is also important to understand the diversity of CAF subtypes in the tumor microenvironment and in spatial proximity to tumor cells. The refinement of knowledge on CAF heterogeneity, and further identification of factors triggering the conversion of tumor-promoting to tumor-restraining CAFs, would provide more specific and personalized therapies for pancreatic cancer patients in the future.

## Figures and Tables

**Table 1:** Origin of cancer-associated fibroblasts (CAFs) and their markers.

Cell Type	Selected Markers/Factors Mentioned in the References	Reference
Pancreatic stellate cells	Desmin, GFAP, acetylcholine receptors	[20]
Mesenchymal stem cell-derived cancer-associated fibroblasts (Gastric cancer)	IL-6, Wnt5a, BMP4	[32]
Bone-marrow derived mesenchymal stem cells (Breast cancer)	CCL5/RANTES	[33]
Cancer-associated mesenchymal stem cells (Pancreatic cancer)	GM-CSF, CD44^+^/CD73^+^/CD90^+^/CD49α^+^	[34]
Adipose-derived mesenchymal stem cells (obese donors) (ovarian cancer)	*ACTA2*, *VEGF*, *FAP*, *S100A4* (FSP), *CSPG4* (NG2)	[35]

**Table 2:** Soluble factors and function.

Soluble Factors	Function	Reference
TGF-β, TNFα, interleukins	Pancreatic stellate cell (PSC) activation	[22]
PDGF, CTGF, EGF	Secreted by activation and transformed PSCs for cancer cell proliferation	[22]
SHH	Produced by pancreatic cancer cells for PSC regulation	[31]
IGF1	Reciprocal signal from SHH-activated PSCs for pancreatic cancer modulation	[31]
IL-6, Wnt5a, BMP4	Produced by mesenchymal stem cell (MSC)-derived cancer-associated fibroblasts (CAFs) and MSCs are recruited to the dysplastic stomach	[32]
CCL5/RANTES	Secreted by bone marrow-derived MSCs (BM-MSCs) to enhance breast cancer motility, invasion, and lung metastasis	[33]
GM-CSF	Secreted by cancer-associated MSCs (CA-MSCs) for pancreatic cancer invasion and metastasis	[34]
IL-6, IL-11, LIF	Secreted by inflammatory CAFs (iCAFs) and activate STAT3 signaling in pancreatic cancer organoids	[36]
IL-1	Secreted by pancreatic cancer cells for inducing LIF and JAK/STAT signaling in iCAFs	[37]

**Table 3:** **Cancer-associated fibroblast** (CAF) subtypes and CAF-subtype-defined markers.

CAF Subtypes	Markers and Factors Expressed in CAF Subtype	Reference	Additional Information
Myofibroblastic CAFs (myCAFs)	*Acta2*, *Vim*, *Ctgf*, *Col1a1*, *Col5a1*, *Col6a1*	[36]	
Myofibroblastic CAFs (myCAFs)	*Tnc*, *Tgfb1*, *Thy1*, *Tagln*, *Col12a1*, *Pdgfrb*	[61]	
Inflammatory CAFs (iCAFs)	*Il1*, *Il6*, *Il11*, *Lif*	[36]	
Inflammatory CAFs (iCAFs)	*Clec3b*, *Col14a1*, *Gsn*, *Ly6c1*, *Cxcl12*	[61]	
CAF subtype A	Periostin/OSF-2	[23]	Shorter overall survival
CAF subtype B	Myosin-11	[23]	Myogenic properties
CAF subtype C	PDPN	[23]	Prolonged overall survival
CAF subtype FB1	*Cxcl14*, *Ptn*, *Igf1*, *Igfbp4*, *Igfbp7*, *Il6*, *Ccl2*, *Ccl7*, *Cxcl12*, *Pdgfra*	[62]	Overlap with iCAFs
CAF subtype FB2	*Ly6a*, *Ly6c1*, *Nov*, *Pi16*	[62]	
CAF subtype FB3	*Lrrn4*, *Gpm6a*, *Nkain4*, *Lgals7*, *Msln*, *Cav1*, *Cdh11*, *Gas6*	[62]	Overlap with myCAFs
Antigen-presenting CAFs (apCAFs)	*Slpi*, *Saa3*, *Cd74*, *H2-Ab1*, *NKain4*, *Irf5*	[61]	
LRRC15-positive CAFs	LRRC15	[63]	Poor response to anti-PD-L1 cancer immunotherapy
Prrx1-positive CAFs	Prrx1	[64]	Involved in CAF plasticity
